# Screening Antioxidant Components in Different Parts of Dandelion Using Online Gradient Pressure Liquid Extraction Coupled with High-Performance Liquid Chromatography Antioxidant Analysis System and Molecular Simulations

**DOI:** 10.3390/molecules29102315

**Published:** 2024-05-15

**Authors:** Xia Cao, Gaoquan Li, Juying Xie, Mengqi Wu, Wenhao Wang, Li Xiao, Zhengming Qian

**Affiliations:** 1 College of Medical Imaging Laboratory and Rehabilitation, Xiangnan University, Chenzhou 423000, China; seasonsyang@sina.cn (X.C.); gaogaokf2024@outlook.com (G.L.); xjy597189746@163.com (J.X.); 2 Key Laboratory of State Administration of Traditional Chinese Medicine, Dongguan HEC Cordyceps R&D Co., Ltd., Dongguan 523850, China

**Keywords:** dandelion, *Taraxacum mongolicum*, antioxidant, high-performance liquid chromatography, mass spectrometry, online gradient pressure liquid extraction, molecular docking

## Abstract

Utilizing online gradient pressure liquid extraction (OGPLE) coupled with a high-performance liquid chromatography antioxidant analysis system, we examined the antioxidative active components present in both the aerial parts and roots of dandelion. By optimizing the chromatographic conditions, we identified the ferric reducing–antioxidant power system as the most suitable for online antioxidant reactions in dandelion. Compared to offline ultrasonic extraction, the OGPLE method demonstrated superior efficiency in extracting chemical components with varying polarities from the samples. Liquid chromatography–mass spectrometry revealed twelve compounds within the dandelion samples, with nine demonstrating considerable antioxidant efficacy. Of these, the aerial parts and roots of dandelion contained nine and four antioxidant constituents, respectively. Additionally, molecular docking studies were carried out to investigate the interaction between these nine antioxidants and four proteins associated with oxidative stress (glutathione peroxidase, inducible nitric oxide synthase, superoxide dismutase, and xanthine oxidase). The nine antioxidant compounds displayed notable binding affinities below −5.0 kcal/mol with the selected proteins, suggesting potential receptor–ligand interactions. These findings contribute to enhancing our understanding of dandelion and provide a comprehensive methodology for screening the natural antioxidant components from herbs.

## 1. Introduction

Dandelion (*Taraxacum mongolicum* Hand. -Mazz.), belonging to the Asteraceae family, is extensively distributed across the Northern Hemisphere [1]. This non-toxic herbaceous plant has both edible and medicinal properties. Various parts of the dandelion, including its leaves, roots, and flowers, are utilized in a range of food products. The young leaves, for instance, are often consumed fresh in salads [2,3,4,5,6,7]. The flowers are used in brewing and dessert preparation [6,7]. Nutritional analysis reveals that dandelion is abundant in minerals, proteins, fibers, vitamins, and balanced trace elements, positioning it as a valuable source of micronutrients [2,3,4,5]. From a medicinal perspective, dandelion boasts a rich tradition in herbal medicine due to its potential benefits such as diuretic, heat relief and detoxification, choleretic, and anti-inflammatory [1,8]. Recently, the antioxidant activity of dandelion has attracted considerable attention. The plant is rich in antioxidant components, including polyphenols and flavonoids, making it a promising natural source for the development of antioxidants [9,10]. Despite the recognition of their health benefits and potential as a reservoir of antioxidant compounds, conventional methodologies to evaluate the antioxidant activity in dandelion are significantly resource-intensive and inefficient in terms of time. Traditional antioxidant assays require substantial resources and a significant time cost, which inherently limits the speed and efficiency of identifying active compounds. In response to the urgent need for a more effective approach, our study is focused on developing a quick and efficient method to screen potential antioxidant compounds in dandelion.

Traditional methods for assessing antioxidant activity, such as 2,2-azino-bis-3-ethylbenzothiazoline-6-sulphonic acid (ABTS), ferric reducing–antioxidant power (FRAP), and 2,2-diphenyl-1-picrylhydrazyl (DPPH) assays, involve reacting with the sample solution and observing the resultant products to determine antioxidant activity. For example, the FRAP method operates by reducing Fe^3+^-ferric tripyridyltriazine (TPTZ) to Fe^2+^-TPTZ under acidic conditions, facilitated by the antioxidant. The absorbance at a wavelength of 593 nm is then measured, serving as an indicator of the total antioxidant capacity of the sample [11].

Lately, integrating high-performance liquid chromatography (HPLC) with antioxidant assessment has become a refined approach for analyzing antioxidants, attributed to its distinct benefits. Offline coupling methods such as ABTS/DPPH-HPLC provide an approach for screening potential antioxidants [12,13]. However, the analysis process can be complex, involving steps such as sample extraction, reaction of the sample with ABTS/DPPH, and HPLC analysis of both the sample solution and the reaction solution. To address these limitations, recent developments have introduced online coupling methods of HPLC and antioxidant evaluation, such as HPLC-DPPH/ABTS/FRAP [14,15,16]. A significant advantage of these methods is their ability to integrate separation and activity evaluation. This integration can effectively bypass some unnecessary analytical steps, thereby saving time. These methods streamline the analysis process, making it more efficient and less labor-intensive, and could be applied in antioxidant research.

Currently, both offline and online HPLC coupling methods for antioxidant evaluation have become valuable tools in rapidly screening antioxidants from natural products. However, despite the elimination of some unnecessary separation steps, the overall process remains time-consuming and labor-intensive, particularly in sample pre-treatment. For the preparation of samples, methods including microwave-assisted procedures, the process of reflux, and sonication techniques are frequently employed in offline contexts. However, considerable quantities of organic solvents, along with extensive extraction periods or substantial sample volumes, are necessitated by such approaches. Additionally, thermal decomposition during the extraction process can decrease the activity of some natural antioxidants. Therefore, while current HPLC-antioxidant evaluation methods have advanced the field significantly, further optimization is necessary to improve efficiency and reduce labor intensity in areas such as sample preparation and extraction. Such improvements could enhance the speed and accuracy of antioxidant analysis in natural products. To address these challenges, an online gradient pressure liquid extraction (OGPLE) combined with an HPLC-based antioxidant assay system was established [17,18]. In this system, a hollow guard cartridge is filled with a powdered sample to form an online extraction cell. The cell is interfaced with an HPLC setup via a valve featuring six ports, utilizing the mobile phase for both extraction and subsequent analytical procedures. Utilizing this system, the extraction of milligram-level samples can be completed within a few minutes, and separation and detection can be achieved in a single run. Additionally, this system forgoes employing any solvents besides the mobile phase, offering a simple, rapid, and efficient green method that minimizes or even eliminates sample handling processes.

Molecular docking serves as a time-efficient and cost-effective computational approach utilized in forecasting the interactions of small molecules with their target proteins. This approach entails positioning a small molecule (referred to as a ligand) within a protein’s binding domain (defined as a receptor), where ongoing modifications in spatial arrangement are conducted to predict the most favorable interaction point and manner of the ligand–receptor ensemble [19]. Molecular docking, a staple in drug discovery efforts, aids in identifying promising lead compounds, enhancing their properties, and forecasting interactions between ligands and proteins [20,21]. While its reliance on computer theoretical simulations often necessitates supplementary experimental validation, many researchers recognize it as a potent instrument for advancing research fields due to its efficiency and predictive capacity [22].

The current study presents the development of an OGPLE combined with an HPLC-based antioxidant assay system for screening the main antioxidant components in the dandelion aerial parts and dandelion roots. Also utilized was the technique of liquid chromatography–tandem mass spectrometry (LC-MS/MS), which pinpointed the compounds specific to antioxidant activity. Subsequently, to investigate the potential interactions between these compounds and oxidative-stress-related proteins, we selected four related proteins, inducible nitric oxide synthase (iNOS), glutathione peroxidase (GSH-Px), superoxide dismutase (SOD), and xanthine oxidase (XOD), for molecular docking analyses to determine their binding affinities. This study aims to elucidate the antioxidant active components of the aerial parts and roots of dandelion, and develop a comprehensive approach for screening antioxidant components from natural products.

## 2. Results and Discussion

### 2.1. Refining Experimental Parameters

#### 2.1.1. Optimization of Chromatographic Conditions

Three chromatographic columns were evaluated in this study: an Agilent ZORBAX SB-AQ column (4.6 mm × 150 mm, 5 μm), a Thermo Hypersil GOLD aQ column (4.6 mm × 150 mm, 5 μm), and a Welch Ultimate AQ-C_18_ column (4.6 mm × 150 mm, 5 μm). Among them, the Thermo Hypersil GOLD aQ column demonstrated superior separation performance and peak shapes (Figure S1). Additionally, the experiment compared three online antioxidant systems: ABTS, FRAP, and DPPH (Figure S2). The results indicated that the FRAP system exhibited distinct antioxidant reaction peaks with a more stable baseline, making it more suitable for online antioxidant reaction systems. In contrast, the ABTS and DPPH systems experienced more significant baseline fluctuations, which are detrimental to the analysis of compound antioxidant activity.

#### 2.1.2. Optimization of OGPLE

**Figure 1:**
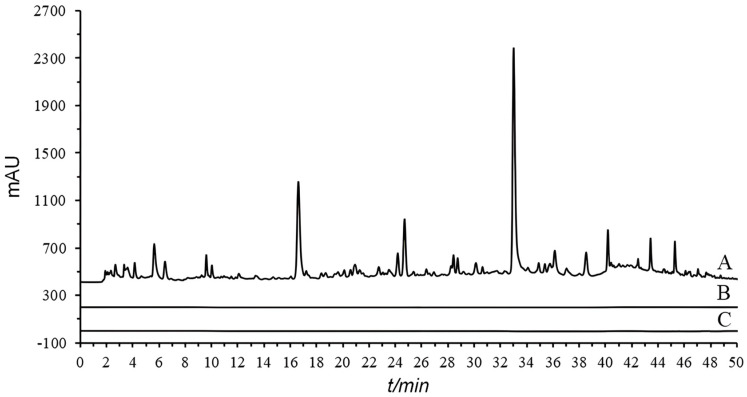
Chromatographic comparison of single and double OGPLE: first OGPLE (A), second OGPLE (B), and blank (C). OGPLE: online gradient pressure liquid extraction.

In this experiment, the types of dispersants and the mixing ratio of sample to dispersant were investigated. By comparing two different dispersants, diatomite and acid-washed diatomite, it was found that the use of acid-washed diatomite resulted in better chromatographic peak shapes and separation performance (Figure S3). The mixing ratios of sample to acid-washed diatomite (1:5, 1:10) were examined (Figure S4). The chromatograms obtained from the 1:5 for sample to acid-washed diatomite showed better response values. Therefore, a mixing ratio of 1:5 was chosen for the OGPLE process. Additionally, a second round of OGPLE was carried out on the samples. The results demonstrated that no chromatographic peaks were observed during the second extraction (Figure 1), suggesting that one OGPLE cycle could effectively obtain all substances present in the samples.

#### 2.1.3. Comparison between OGPLE and Offline Extraction

**Figure 2:**
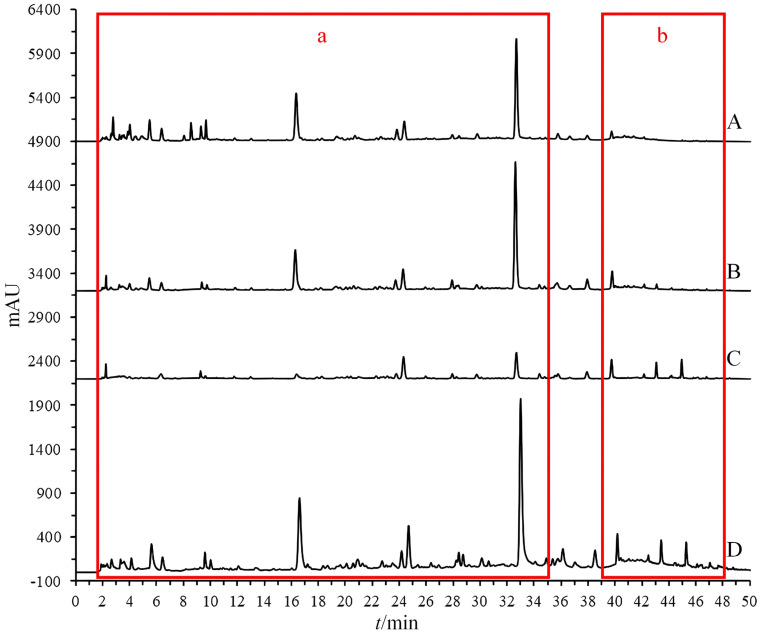
Chromatographic comparison of offline and online extraction methods (water extraction (A), 50% methanol extraction (B), anhydrous methanol extraction (C), and OGPLE (D)) with high-polarity components (a) and low-polarity components (b). OGPLE: online gradient pressure liquid extraction.

During the experimental process, it was discovered that employing offline ultrasonic extraction methods proved challenging for the simultaneous extraction of chemical components with different polarities from dandelion. The experiments utilized three solvents—water, 50% methanol, and anhydrous methanol—for extracting the samples (Figure 2). The results indicated that certain low-polarity components in the extracts obtained with water and 50% methanol were missing (Figure 2b), while extracts obtained with anhydrous methanol were missing some high-polarity components (Figure 2a). This study employed the OGPLE method, which utilizes a gradient alteration in mobile phase polarity for sample extraction. This gradient shifted from high to low polarity, meeting the extraction conditions for compounds of different polarities present in the sample. This approach enabled the maximum extraction of components within a single analysis, significantly saving on analysis time and solvent consumption.

The stability of the aqueous ultrasound extract of dandelion was analyzed in 24 h. Both caffeic acid and cichoric acid exhibited a relative standard deviation (RSD) greater than 10%, with caffeic acid increasing by 38.1% and cichoric acid decreasing by 29.2% after 24 h. This indicates that the aqueous extract of dandelion is not stable. In contrast, the OGPLE method used in this study allows the compounds to be extracted by the mobile phase and immediately analyzed by HPLC. This reduces the decomposition and reaction of compounds, accurately reflecting the content of various components in the sample and enhancing the accuracy of the analysis.

### 2.2. Method Validation

This experiment investigated the specificity of the method by analyzing blank solutions, standard solutions, and dandelion aerial part sample solutions (Figure S5). The method demonstrated good chromatographic peak separation with no interference from impurity peaks at the elution positions, allowing for the qualitative analysis of the samples. Furthermore, following the procedure outlined in the “OGPLE of Sample” Section, three dandelion aerial part samples were extracted online. The findings demonstrated that the RSD for the principal chromatographic peaks across all three samples remained below 10%, indicating that the established method has good repeatability.

### 2.3. Identification and Quantification of Antioxidant Active Components from Dandelion Aerial Parts and Dandelion Roots

**Figure 3:**
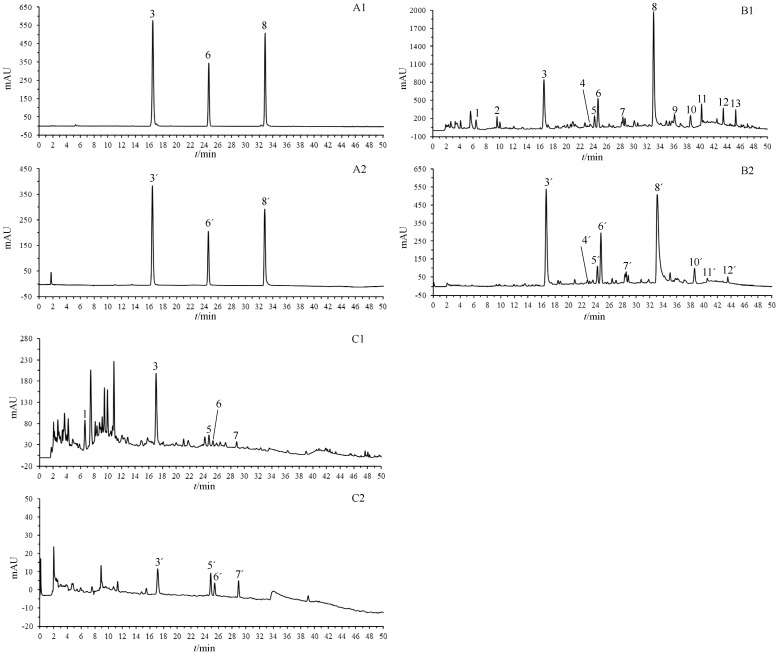
OGPLE coupled with HPLC antioxidant analysis system for dandelion aerial part and dandelion root samples. (**A**) Standard solution; (**B**) dandelion aerial parts; (**C**) dandelion roots. (**A1**,**B1**,**C1**) Total compound chromatograms. (**A2**,**B2**,**C2**) Fe^2+^-TPTZ peaks detected after reaction with FRAP solution. Peak Assignments: 1. uric acid, 2. adenosine, 3. caftaric acid, 4. aesculetin, 5. neochlorogenic acid, 6. caffeic acid, 7. caffeoylmalic acid, 8. cichoric acid, 9. luteolin 7-O-glucoside, 10. 3,5-Di-caffeoylquinic acid, 11. phlorizin, 12. luteolin, 13. unknown. OGPLE: online gradient pressure liquid extraction; HPLC: high-performance liquid chromatography; TPTZ: ferric tripyridyltriazine; FRAP: ferric reducing–antioxidant power.

In this investigation, OGPLE combined with the HPLC-based antioxidant assay system was employed for the separation and subsequent online antioxidant analysis of compounds in the dandelion aerial part and dandelion root samples (Figure 3). The analysis included measuring compound retention times at two distinct wavelengths: initially at 275 nm (Detector 1) to detected the compounds, followed by post-reaction detection at 593 nm (Detector 2) to measure the absorbance of the Fe^2+^-TPTZ complex. This phase enabled linking specific substances with their antioxidative actions, determined by analyzing the chromatographic peaks observed pre- and post-reaction. It was found that nine compounds demonstrated antioxidant activity in the dandelion aerial part samples (Figure 3(B2)) and four in the dandelion root samples (Figure 3(C2)).

MS was used to identify compounds in the dandelion aerial part and root samples (Table 1). Twelve compounds were identified in the dandelion aerial part samples, namely uric acid (1), adenosine (2), caftaric acid (3), aesculetin (4), neochlorogenic acid (5), caffeic acid (6), caffeoylmalic acid (7), cichoric acid (8), luteolin 7-O-glucoside (9), 3,5-di-caffeoylquinic acid (10), phlorizin (11), and luteolin (12). In the dandelion root samples, five compounds were identified: uric acid (1), caftaric acid (3), neochlorogenic acid (5), caffeic acid (6), and caffeoylmalic acid (7). The identification was based on the retention times of the reference components, as well as MS data with known studies and databases [23,24,25,26,27].

Within the dandelion aerial part samples, the compounds exhibiting antioxidant activity included caftaric acid (3), aesculetin (4), neochlorogenic acid (5), caffeic acid (6), caffeoylmalic acid (7), cichoric acid (8), 3,5-di-caffeoylquinic acid (10), phlorizin (11), and luteolin (12). For the dandelion root samples, the antioxidant active components were identified as caftaric acid (3), neochlorogenic acid (5), caffeic acid (6), and caffeoylmalic acid (7).

The quantitative analysis of nine antioxidant active compounds in the aerial parts and roots of the dandelion samples was carried out (Table S1). Caffeic acid content was measured by employing its calibration curve (*y* = 63.85*x* − 30.831; *R*^2^ = 0.9995) of the standard. Concurrently, the concentrations of the other eight compounds were estimated by the caffeic acid calibration curve as a reference, owing to the absence of standards for these compounds. This surrogate approach, utilized in previous research, is a common method in the absence of specific standard substances [28]. The quantitative analysis of the dandelion revealed that caftaric acid (1.24 mg/g dry weight [DW], Percentage: 22%), caffeic acid (0.478 mg/g DW, Percentage: 8%), and cichoric acid (2.75 mg/g DW, Percentage: 48%) were the major antioxidants in the aerial parts, with caftaric acid (0.205 mg/g DW, Percentage: 78%) being the primary antioxidant in the roots. Previous research has indicated that caftaric acid and caffeic acid in plant extraction remain stable for over 70 days at room temperature [29], while cichoric acid is stable for more than 30 days in dry, light-protected conditions [30]. The addition of these antioxidant compounds into food products may prolong storage duration [31]. Consequently, it can be considered that the antioxidant active compounds in dandelion products are highly stable. In addition, the quantitative analysis results showed that the content of antioxidants in the aerial portion is higher than in the root. To confirm this finding, an offline antioxidant analysis was conducted, and the result indicates that the antioxidant ability of the dandelion aerial portions is more than 4 times that of the roots (Figure S6). This result is consistent with the quantitative analysis of antioxidants in dandelion. These findings align with previous studies [32], demonstrating the reliability of online antioxidant analysis as a valid method. It is important to acknowledge that the reported findings are preliminary, and a thorough evaluation of the antioxidant capacity of these identified compounds would necessitate detailed quantitative analyses along with functional assays. Future research endeavors should strive to accurately quantify these compounds in dandelion while investigating their synergistic effects and bioavailability. This study will help to improve our knowledge of the potential antioxidant efficacy of dandelion, and improve the quality evaluation and product development of dandelion.

### 2.4. Molecular Docking Analysis

**Figure 4:**
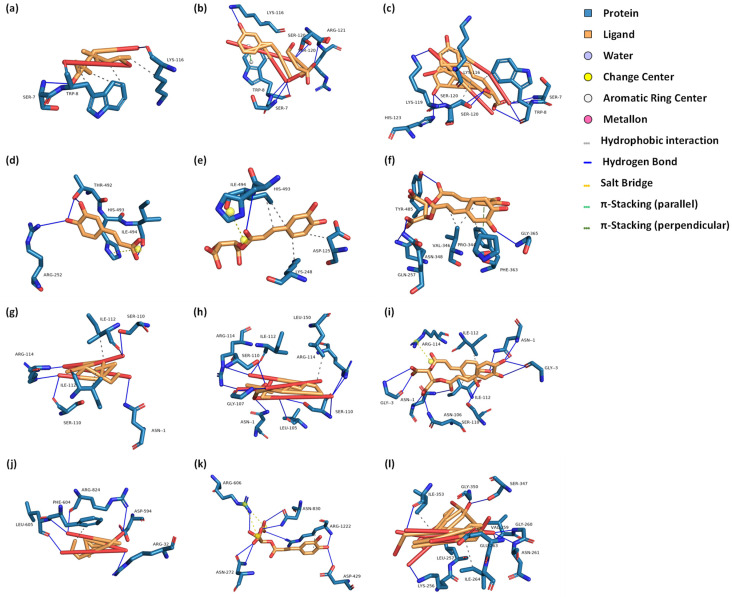
Docking conformations of dandelion’s principal antioxidant compounds with GSH-Px, iNOS, SOD, and XOD receptors. (**a**–**c**) Interactions with the GSH-Px receptor for caffeic acid, caftaric acid, and cichoric acid, respectively. (**d**–**f**) Interactions with the iNOS receptor for caffeic acid, caftaric acid, and cichoric acid, respectively. (**g**–**i**) Interactions with the SOD receptor for caffeic acid, caftaric acid, and cichoric acid, respectively. (**j**–**l**) Interactions with the XOD receptor for caffeic acid, caftaric acid, and cichoric acid, respectively. GSH-Px: glutathione peroxidase; iNOS: inducible nitric oxide synthase; SOD: superoxide dismutase; XOD: xanthine oxidase.

In the field of antioxidant research, molecular docking analysis has been widely adopted, particularly for exploring how antioxidants interact with specific targets to elicit their functions [33,34]. This approach not only aids in understanding the molecular-level efficacy of antioxidants, but also lays a solid theoretical foundation for online antioxidant screening. Molecular docking analysis was conducted to explore the interaction dynamics of nine promising antioxidant compounds with four proteins associated with oxidative stress, including GSH-Px, iNOS, SOD, and XOD (Table 2). The docking conformations and key binding residues of dandelion’s principal antioxidant compounds in the interaction with receptors were also simulated and delineated (Figure 4; Table 3). Typically, an affinity score below −5.0 kcal/mol indicates receptor–ligand coupling [35]. The results indicate that all nine antioxidant compounds exhibit affinity scores below −5.0 kcal/mol with the four proteins. GSH-Px, an enzyme capable of peroxide clearance [36], demonstrated affinity scores of −5.3 kcal/mol for caftaric acid, −5.4 kcal/mol for caffeic acid, and −6.2 kcal/mol for cichoric acid. iNOS, responsible for nitric oxide production and the modulation of inflammation and protective stress responses [37], demonstrated affinity scores of −6.8 kcal/mol for caftaric acid, −6.8 kcal/mol for caffeic acid, and −9.4 kcal/mol for cichoric acid. SOD, known for its antioxidant properties [38], demonstrated affinity scores of −7.1 kcal/mol for caftaric acid, −6.2 kcal/mol for caffeic acid, and −7.5 kcal/mol for cichoric acid. XOD, involved in generating reactive oxygen species [39], demonstrated affinity scores of −8.5 kcal/mol for caftaric acid, −7.0 kcal/mol for caffeic acid, and −8.8 kcal/mol for cichoric acid. These docking results highlight the significant binding affinities of the nine compounds with the selected receptors, offering insights into their antioxidant effects and other potential biological activities. Of course, these findings will need further validation through experimental studies. In conclusion, the molecular docking analysis revealed significant binding affinities of the nine compounds with the selected receptors, shedding light on their antioxidant effects and potential biological activities.

## 3. Materials and Methods

### 3.1. Chemicals and Reagents

Ultrapure water was produced in a Milli-Q Advantage A10 water purification system (Merck KGaA., Darmstadt, Hesse, Germany). Acetic acid (HPLC grade), formic acid (HPLC grade), 2,2’-azino-bis(3-ethylbenzothiazoline-6-sulfonic acid) diammonium salt (98%), 2,4,6-tris(2-pyridyl)-s-triazine (99%), and potassium persulfate (Analytical Reagent) were obtained from Shanghai Aladdin Biochemical Technology Co., Ltd. (Shanghai, China). Ferric chloride (Chemically Pure) was obtained from Shanghai Macklin Biochemical Co., Ltd. (Shanghai, China). Sodium acetate anhydrous (Analytical Reagent) was obtained from Guangdong Guanghua Sci-Tech Co., Ltd. (Shantou, Guangdong, China). Celatom^®^, acid-washed and 2,2-diphenyl-1-picrylhydrazyl (100%) were obtained from Sigma-Aldrich, Inc. (Merck KGaA., Darmstadt, Hesse, Germany). Anhydrous methanol (Analytical Reagent) was obtained from Fuchen (Tianjin) Chemical Reagent Co., Ltd. (Tianjin, China). Acetonitrile (HPLC grade) was obtained from ANPEL Laboratory Technologies (Shanghai) Inc. (Shanghai, China). Ethanol (HPLC grade) was obtained from Krude Company, Inc. (Los Angeles, CA, USA). Caffeic acid (98.7%) was obtained from Shanghai Standard Technology Co., Ltd. (Shanghai, China). Hydrochloric acid (Analytical Reagent), phosphorus pentoxide (Analytical Reagent), and sodium hydroxide (Analytical Reagent) were obtained from Chengdu Kelong Chemical Co., Ltd. (Chengdu, Sichuan, China). Caftaric acid (98.8%) and cichoric acid (91.0%) were prepared in our laboratory.

### 3.2. Sample of Dandelion

The dandelion aerial part samples were procured from Bozhou Jingwan Chinese MEDICINE FACTORY (Bozhou, Anhui, China), with the origin being Bozhou, Anhui, China. The dandelion root samples were obtained from KANGMEI (Bozhou) HUATUO International Chinese MEDICINE City Co., Ltd. (Bozhou, Anhui, China), with the origin being Changbai Mountain in Jilin, China.

### 3.3. Standard Solution Preparation

A precise measure of standard substances, specifically caffeic acid, caftaric acid, and cichoric acid, was utilized to concoct a combined standard solution at an optimal concentration with 50% methanol. Following preparation, this solution underwent filtration with a 0.22 μm organic membrane filter and was preserved at a refrigerated temperature of 4 °C.

### 3.4. Preparation of Antioxidant Assay Reagents

#### 3.4.1. FRAP Solution

Preparation of ferric chloride solution (20 mmol/L): We weighed out 652.6 mg of ferric chloride and dissolved it in 200 mL of ultrapure water, mixing thoroughly.

Preparation of TPTZ solution (10 mmol/L): We weighed out 624.7 mg of 2,4,6-tris(2-pyridyl)-s-triazine and dissolved it in 200 mL of 40 mmol/L hydrochloric acid, mixing thoroughly.

Preparation of sodium acetate–acetic acid buffer (300 mmol/L): We weighed 1.8225 g of anhydrous sodium acetate, dissolved it in 1 L of ultrapure water, then add 16 mL of acetic acid, and mix. We adjusted the pH to 3.6 using 1 mol/L sodium hydroxide or hydrochloric acid.

We combined the aforementioned solutions in a ratio of 1:1:10 (ferric chloride solution/TPTZ solution/sodium acetate–acetic acid buffer).

#### 3.4.2. ABTS Solution

We weighed out 194.1 mg of 2,2’-azino-bis (3-ethylbenzothiazoline-6-sulfonic acid) diammonium salt and 68.6 mg of potassium persulfate, and dissolved them in ultrapure water (100 mL) to prepare a mixed solution with concentrations of 3.5 mmol/L for 2,2’-azino-bis(3-ethylbenzothiazoline-6-sulfonic acid) diammonium salt and 2.5 mmol/L for potassium persulfate. The reaction took place in the dark at 4 °C for 12 h. Prior to usage, we diluted the sample with ethanol and adjusted the solution to an absorbance of 1.0 at 750 nm on a Cary 60 UV-Vis Spectrophotometer (Agilent Technologies, Inc., Santa Clara, CA, USA).

#### 3.4.3. DPPH Solution

To prepare a DPPH solution of 0.2 mmol/L concentration, we measured 24.2 mg of 2,2-diphenyl-1-picrylhydrazyl, which was then completely dissolved in 300 mL of anhydrous methanol.

### 3.5. Offline Extraction of Sample

Referencing previous methodologies [40], we weighed approximately 0.5 g of the sample powder three times, and placed each portion into a separate 15 mL centrifuge tube. To each tube, we added 5 mL of ultrapure water, 50% methanol, and anhydrous methanol, respectively. The mixture was subjected to ultrasonic extraction for 30 min (at a power of 380 W and a frequency of 37 kHz), followed by thorough mixing. We collected the supernatant and filtered it through a 0.22 μm membrane. The filtrate obtained was used as the sample solution.

### 3.6. OGPLE of Sample

**Figure 5:**
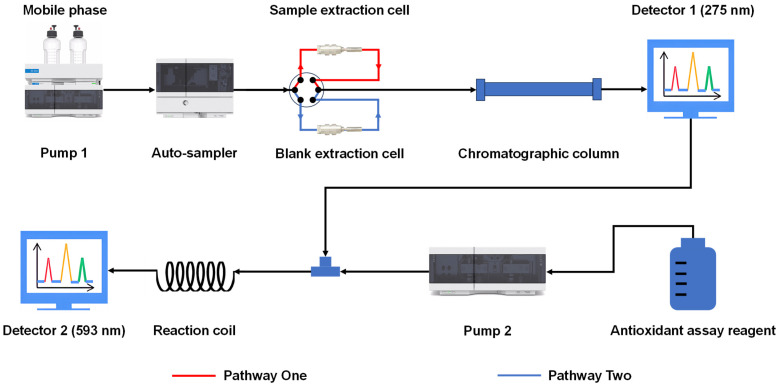
Schematic of the OGPLE-HPLC antioxidant analysis system. OGPLE: online gradient pressure liquid extraction; HPLC: high-performance liquid chromatography.

The OGPLE combined with the HPLC-based antioxidant assay system was constructed based on previous studies (Figure 5) [17,18]. The sample powder was combined with acid-washed diatomite at a 1:5 ratio. Subsequently, approximately 5.0 mg of the homogeneous mixture was measured accurately and loaded into an empty SecurityGuard Standard, and the void was filled with acid-washed diatomite and sealed securely at both ends with a 0.22 μm filter membrane. It was then loaded back into the SecurityGuard Cartridge (3.0 × 4.0 mm; Phenomenex, Inc., Torrance, CA, USA) to form a sample extraction cell. The assembled sample extraction cell and a blank extraction cell were connected to the LC system via a six-way valve, utilizing the mobile phase for the online micro-extraction process. When the sample was analyzed, the six-port valve transitioned to the sample extraction cell to facilitate online extraction. When replacing the sample became necessary, the valve was adjusted to the blank extraction cell, thereby efficiently purifying the system and preparing it for the introduction of a new sample.

### 3.7. HPLC Instruments and Conditions

The chromatographic analysis in this study was carried out using an Agilent 1260 Infinity LC System (Agilent Technologies, Inc.). The chromatographic column employed was a Thermo Hypersil GOLD aQ column (4.6 mm × 150 mm, 5 μm) maintained at a temperature of 25 °C. The composition of the mobile phase was 0.1% formic acid (A) and acetonitrile (B), with the gradient elution described as follows: initiating with 0% B from 0 to 3 min; a linear escalation to 4% B from 3 to 7 min; a rise to 5% B between 7 and 11 min; an increment to 10% B from 11 to 20 min; a progressive increase to 18% B from 20 to 30 min; an elevation to 21% B between 30 and 36 min; culminating in a surge to 70% B from 36 to 50 min. The flow rate was set at 1 mL/min, with the detection wavelength at 275 nm. A volume of injection of 5 μL was used for offline extraction analysis without additional sample introduction for online micro-extraction analysis.

### 3.8. Offline Antioxidant Analysis

Measurements were conducted using a BioTek Synergy H1 Multimode Reader (Agilent Technologies, Inc.). Dandelion sample offline extraction solutions (50% methanol extraction) were diluted fivefold for aerial parts and twofold for roots. Concurrently, solutions of the caffeic acid standard were created, with concentrations measuring 19.8, 99.0, 198, 297, and 396 μg/mL. Assays were set up as follows: a blank group (200 μL FRAP solution + 10 μL blank solvent); a sample group (200 μL FRAP solution + 10 μL sample solution); and a reference compound group (200 μL FRAP solution + 10 μL caffeic acid standard solution of varying concentrations). These mixtures were gently combined in a 96-well plate, incubated at room temperature for 5 min, agitated on a plate shaker for 1 min, and then measured for absorbance at 593 nm. To quantify the antioxidative capacity of the samples, the absorbance readings from the sample extracts were calculated against the standard curve generated from the caffeic acid standards. The antioxidant power was expressed in terms of caffeic acid equivalents, providing a comparative measure of antioxidative strength across different samples.

### 3.9. Online Antioxidant Analysis

As depicted in Figure 5, after separation through HPLC, the sample solution was combined with antioxidant assay reagents. These reagents were administered by another HPLC pump at a delivery rate of 0.5 mL/min. The mixture then reacted within a 1.5 m × 0.25 μm Polyether ether ketone tube before entering the ultraviolet detector for the assessment of antioxidant components. These components were detected at wavelengths of 593 nm (FRAP), 750 nm (ABTS), and 517 nm (DPPH).

### 3.10. Preparation of Caftaric Acid and Cichoric Acid

The preparation of standard substances for caftaric acid and cichoric acid was carried out on the Agilent 1260 Infinity HPLC System (Agilent Technologies, Inc.). Sample extracts were separated on an Agilent ZORBAX SB-C_18_ column (9.4 mm × 250 mm, 5 µm) with an injection volume of 100 µL and a flow rate of 4 mL/min. The elution gradient and mobile phase followed the protocol described in the “HPLC Instruments and Conditions” Section. Eluates for caftaric acid (16 to 17 min) and cichoric acid (33 to 34 min) were collected across five consecutive injections, pooled separately for each compound, and then subjected to vacuum drying. The drying process involved placing Petri dishes containing phosphorus pentoxide along with another dish containing the eluates inside a vacuum oven (Model DZF6050; Gongyi Yuhua Instrument Co., Ltd., Gongyi, Henan, China), depressurizing for 30 min and then drying continuously at room temperature (25 ± 5 °C) for 15 h. The residues were then re-dissolved in 50% methanol to obtain caftaric acid crude extract solution and cichoric acid crude extract solution.

The extracted solutions underwent refinement using a Thermo Hypersil GOLD aQ column (4.6 mm × 150 mm, 5 μm), employing a mobile phase consisting of 0.1% formic acid (A) and acetonitrile (B). Specifically, for the caftaric acid raw extract, a gradient elution strategy was utilized that started with 4% B for 0 to 16 min; increased to 10% B from 16 to 18 min; and was then held constant at 10% B for 18 to 23 min. The procedure involved an injection volume of 80 µL and a flow rate set at 1 mL/min. The eluate collected between 13 and 14 min was vacuum-dried following the previous method, yielding the caftaric acid standard. Similarly, the cichoric acid crude extract solution was processed with an isocratic elution of 14% B for 20 min, with an injection volume of 80 µL and a flow rate of 1 mL/min. The eluate collected between 14 and 15 min was also vacuum-dried, resulting in the cichoric acid standard.

### 3.11. LC-MS/MS Qualitative Analysis Conditions

Chromatographic separation utilized the Vanquish Flex UHPLC System (Thermo Fisher Scientific Inc., Franklin, MA, USA), and mass spectrometric analysis was conducted using a Q Exactive Focus Orbitrap LC-MS/MS System (Thermo Fisher Scientific Inc.). The LC conditions are described in the “HPLC Instruments and Conditions” Section. Performing in both the positive and negative ion states, the MS analysis used full scan and dd-MS^2^ scanning modes. The comprehensive scan extended across a mass-to-charge (*m/z*) range of 100 to 1500, acquiring data at a resolution of 70,000. For dd-MS^2^ scans, the scope was set from *m/z* 50 to 1000, with a resolution setting of 17,500. Normalized collision energies were programmed in a stepped sequence of 10, 20, and 40. The ionization method employed was heated electrospray ionization (H-ESI), characterized by sheath, auxiliary (Aux), and sweep gas flow rates of 60 arb, 20 arb, and 3 arb, respectively. The spray voltage was uniformly maintained at 3500 V across both positive and negative modes. Additionally, the capillary and Aux gas heaters were regulated at temperatures of 400 °C and 450 °C, respectively.

### 3.12. Quantitative Analysis of Antioxidant Compounds

Adhering to our previously described “Standard Solution Preparation” Section, caffeic acid standard solutions were prepared with concentrations of 1.98 μg/mL, 99.0 μg/mL, 198 μg/mL, 396 μg/mL, and 594 μg/mL. Samples underwent extraction per the method outlined in the “OGPLE of Sample” Section, and the HPLC analysis followed the protocols outlined in the “HPLC Instruments and Conditions” Section. The calculation of the content of all antioxidant compounds in the sample was based on the caffeic acid standard curve.

### 3.13. Molecular Docking Study

Using the PyMOL (https://pymol.org/) and AutoDock Vina 1.2.0 software tools, this study primarily conducted molecular docking [41,42,43]. This experimental process necessitates the three-dimensional structure of the ligand and the crystal structure of the receptor. To achieve this, the three-dimensional structures of the antioxidants were created utilizing the Chem3D version 20.0.0.41 software (PerkinElmer Inc., Shelton, CT, USA). The crystallographic configurations for pertinent proteins, such as GSH-Px (PDB ID: 2WGR), iNOS (PDB ID: 1M8D), SOD (PDB ID: 1TO4), and XOD (PDB ID: 1FIQ), were acquired from the RCSB Protein Data Bank [44]. Subsequently, the receptor protein underwent processing utilizing the AutoDock Vina and PyMOL software, which included dehydration, removal of the ligand, and the addition of charges. The AutoDock Vina software was then employed to frame the entire receptor protein by adjusting the coordinates, length, width, and height of the Grid Box, and the parameters were recorded accordingly. Ultimately, the AutoDock Vina software was used to conduct molecular docking of the receptor and ligand, generating a maximum of 100 models, and the affinity results were obtained. The concluding step involved analyzing and visualizing the docking results with the lowest binding energies using PyMOL, and the binding site residues were identified through the Protein–Ligand Interaction Profiler [45].

## 4. Conclusions

OGPLE combined with the HPLC-based antioxidant assay system established in this study significantly enhances the rapid identification of potentially health-beneficial compounds within dandelion aerial part and dandelion root samples. This approach specializes in rapidly detecting antioxidants, providing valuable information for further research. Additionally, the molecular docking evaluations demonstrated the binding affinity of these compounds with the chosen receptors, illuminating their prospective antioxidant properties and various biological activities. In summary, the combined approach of OGPLE and the HPLC-based antioxidant assay, complemented by molecular docking analysis, offers an effective framework for the quick identification and validation of antioxidants in plant-based materials. This approach serves as a valuable tool in antioxidant research, facilitating the use of natural compounds for health-related purposes.

## Figures and Tables

**Table 1:** MS data of components in the aerial parts and roots of dandelion.

Peak No.	Compound	RT	Molecular Formula	Exact Mass	Adduct Ion	Precursor Ion	Product Ion	Reference
1	Uric acid	6.453	C_5_H_4_N_4_O_3_	168.0283	[M-H]^−^	167.0200	124.0141, 96.0190	[23]
2	Adenosine	9.593	C_10_H_13_O_4_N_5_	267.0968	[M+H]^+^	268.1042	136.0620	[24]
3	Caftaric acid	16.606	C_13_H_12_O_9_	312.0481	[M-H]^−^	311.0411	179.0343, 149.0083, 135.0442	[25]
4	Aesculetin	23.506	C_9_H_6_O_4_	178.0266	[M-H]^−^	177.0183	149.0236, 133.0284, 105.0334	[23]
5	Neochlorogenic acid	24.173	C_16_H_18_O_9_	354.0951	[M-H]^−^	353.0879	191.0555	[24]
6	Caffeic acid	24.693	C_9_H_8_O_4_	180.0423	[M-H]^−^	179.0340	135.0441, 133.0283	[26]
7	Caffeoylmalic acid	28.419	C_13_H_12_O_8_	296.0532	[M-H]^−^	295.0460	179.0343, 135.0441, 133.0132, 115.0025	[27]
8	Cichoric acid	33.006	C_22_H_18_O_12_	474.0798	[M-H]^−^	473.0728	311.0412, 293.0306, 179.0342, 149.0082, 135.0040	[26]
9	Luteolin 7-O-glucoside	36.132	C_21_H_20_O_11_	448.1006	[M-H]^−^	447.0940	285.0407	[26]
10	3,5-Di-caffeoylquinic acid	38.519	C_25_H_24_O_12_	516.1268	[M-H]^−^	515.1201	353.0886, 191.0556, 179.0343	[26]
11	Phlorizin	40.179	C_21_H_24_O_10_	436.1370	[M-H]^−^	435.1303	273.0775, 167.0342	[24]
12	Luteolin	43.425	C_15_H_10_O_6_	286.0477	[M-H]^−^	285.0405	175.0395, 151.0028, 133.0285	[23]
13	Unknown	45.279	-	-	[M-H]^−^	329.2335	229.1445, 211.1338, 199.4364, 171.1018	-

RT: retention time; MS: mass spectrometry.

**Table 2:** Parameters and outcomes of molecular docking analysis.

Receptor	Number of Points	Center Grid Box	Spacing	Ligand	Affinity (kcal/mol)
GSH-Px	X-dimension = 104 Y-dimension = 96 Z-dimension = 126	X center = 12.253 Y center = 8.525 Z center = 14.137	0.375	Caftaric acid	−5.3
				Aesculetin	−5.4
				Neochlorogenic acid	−6.9
				Caffeic acid	−5.4
				Caffeoylmalic acid	−6.3
				Cichoric acid	−6.2
				3,5-Di-caffeoylquinic acid	−7.3
				Phlorizin	−5.9
				Luteolin	−6.8
iNOS	X-dimension = 82 Y-dimension = 102 Z-dimension = 126	X center = 124.075 Y center = 110.548 Z center = 61.561	0.914	Caftaric acid	−6.8
				Aesculetin	−7.5
				Neochlorogenic acid	−7.9
				Caffeic acid	−6.8
				Caffeoylmalic acid	−7.3
				Cichoric acid	−9.4
				3,5-Di-caffeoylquinic acid	−8.3
				Phlorizin	−9.1
				Luteolin	−9.5
SOD	X-dimension = 56 Y-dimension = 126 Z-dimension = 110	X center = 11.495 Y center = 36.951 Z center = 32.456	1.000	Caftaric acid	−7.1
				Aesculetin	−6.3
				Neochlorogenic acid	−8.1
				Caffeic acid	−6.2
				Caffeoylmalic acid	−7.0
				Cichoric acid	−7.5
				3,5-Di-caffeoylquinic acid	−7.9
				Phlorizin	−8.5
				Luteolin	−8.5
XOD	X-dimension = 88 Y-dimension = 92 Z-dimension = 72	X center = 23.556 Y center = 32.646 Z center = 101.417	1.000	Caftaric acid	−8.5
				Aesculetin	−7.3
				Neochlorogenic acid	−8.3
				Caffeic acid	−7.0
				Caffeoylmalic acid	−7.7
				Cichoric acid	−8.8
				3,5-Di-caffeoylquinic acid	−9.8
				Phlorizin	−9.2
				Luteolin	−9.8

iNOS: inducible nitric oxide synthase; GSH-Px: glutathione peroxidase; SOD: superoxide dismutase; XOD: xanthine oxidase.

**Table 3:** The key binding residues of three major antioxidants.

Receptor	Ligand	Binding Site Interactions	Key Residues in Interaction
GSH-Px	Caftaric acid	Hydrophobic interactions	TRP8A
		Hydrogen bonds	SER7A, TRP8A, LYS116A, SER120A, ARG121A
		π-stacking	TRP8A
	Caffeic acid	Hydrophobic interactions	TRP8A, LYS116A
		Hydrogen bonds	SER7A, TRP8A, LYS116A
	Cichoric acid	Hydrophobic interactions	TRP8A, LYS116A, SER120A
		Hydrogen bonds	SER7A, TRP8A, LYS119A, SER120A, HIS123A
iNOS	Caftaric acid	Hydrophobic interactions	ASP125B, LYS248B, ILE494B
		Hydrogen bonds	ILE494B
		Salt bridges	HIS493B
	Caffeic acid	Hydrophobic interactions	HIS493A
		Hydrogen bonds	ARG252A, THR492A, ILE494A
		Salt bridges	HIS493A
	Cichoric acid	Hydrophobic interactions	PRO344A, VAL346A
		Hydrogen bonds	GLN257A, ASN348A, GLY365A, TYR485A
		π-Stacking	PHE363A
SOD	Caftaric acid	Hydrophobic interactions	LEU150B
		Hydrogen bonds	ASN-1A, LEU105A, GLY107B, SER110A, SER110B, ILE112B, ARG114A, ARG114B
	Caffeic acid	Hydrophobic interactions	ILE112A, ILE112B
		Hydrogen bonds	ASN-1B, SER110A, SER110B, ILE112A, ARG114B
	Cichoric acid	Hydrophobic interactions	ILE112C, ILE112D
		Hydrogen bonds	GLY-3C, GLY-3D, ASN-1C, ASN-1D, ASN106D, SER110D, ILE112D
		Salt bridges	ARG114C
XOD	Caftaric acid	Hydrophobic interactions	ARG1222C
		Hydrogen bonds	ASN272B, ASP429B, ARG606C, ASN830C, ARG1222C
		Salt bridges	ARG606C
	Caffeic acid	Hydrophobic interactions	PHE604C
		Hydrogen bonds	ARG32A, ASP594C, LEU605C, ARG824C
	Cichoric acid	Hydrophobic interactions	LEU257B, GLU263B, ILE264B, ILE353B
		Hydrogen bonds	LYS256B, VAL259B, GLY260B, ASN261B, SER347B, GLY350B, ILE353B

iNOS: inducible nitric oxide synthase; GSH-Px: glutathione peroxidase; SOD: superoxide dismutase; XOD: xanthine oxidase.

## Data Availability

All data, models, and code generated or used during this study are presented in the submitted article.

## Supplementary Materials

The following supporting information can be downloaded at: https://www.mdpi.com/article/10.3390/molecules29102315/s1, Figure S1: Comparative chromatograms: Thermo Hypersil GOLD aQ (4.6 mm × 150 mm, 5 μm) (A), Agilent ZORBAX SB-AQ (4.6 mm × 150 mm, 5 μm) (B), and Welch Ultimate AQ-C_18_ (4.6 mm × 150 mm, 5 μm) (C); Figure S2: Comparison of chromatograms from online antioxidant systems: ferric reducing–antioxidant power (FRAP) (A), 2,2-azino-bis-3-ethylbenzothiazoline-6-sulphonic acid (ABTS) (B), and 2,2-diphenyl-1-picrylhydrazyl (DPPH) (C); Figure S3: Chromatograms of different dispersants: acid-washed diatomite (A) and diatomite (B); Figure S4: Chromatograms of different ratios (dandelion aerial part to acid-washed diatomite); Figure S5: Chromatograms of dandelion aerial parts (A), standard solution (B), and blank solution (C). Figure S6: Offline antioxidant analysis of dandelion aerial parts and roots (*n* = 3; mean ± standard deviation). Aerial parts: 86.1 mg/g; roots: 18.3 mg/g. *** *p* < 0.001 versus the roots, evaluated using Student’s *t*-test. Table S1: The contents of nine antioxidant compounds in the aerial parts and roots of dandelion (*n* = 3).
