# Supplementary material for: Screening Antioxidant Components in Different Parts of Dandelion Using Online Gradient Pressure Liquid Extraction Coupled with High-Performance Liquid Chromatography Antioxidant Analysis System and Molecular Simulations
Source: Molecules. 2024 May 15;29(10):2315. doi: 10.3390/molecules29102315 (PMC11124315; doi:10.3390/molecules29102315)
Supplement: Supplementary file 1 [file molecules-29-02315-s001.zip › molecules-2966015-supplementary.pdf]

Supplementary Material

Table S1. The contents of nine antioxidant compounds in the aerial parts and roots of dandelion (*n* = 3).

Compound	Aerial part		Root	
	Content (mg/g DW)	Percentage (%)	Content (mg/g DW)	Percentage (%)
Caftaric acid*	1.24	22%	0.205	78%
Aesculetin	0.0939	2%	-	-
Neochlorogenic acid	0.203	4%	0.0276	10%
Caffeic acid*	0.478	8%	0.0155	6%
Caffeoylmalic acid	0.151	3%	0.0157	6%
Cichoric acid*	2.75	48%	-	-
3,5-Di-caffeoylquinic acid	0.236	4%	-	-
Phlorizin	0.354	6%	-	-
Luteolin	0.187	3%	-	-

DW: dry weight. *: the major antioxidant.

$\text{Percentage (\%)} = (\text{Individual compound content}/\text{Sum of all compound contents}) \times 100$

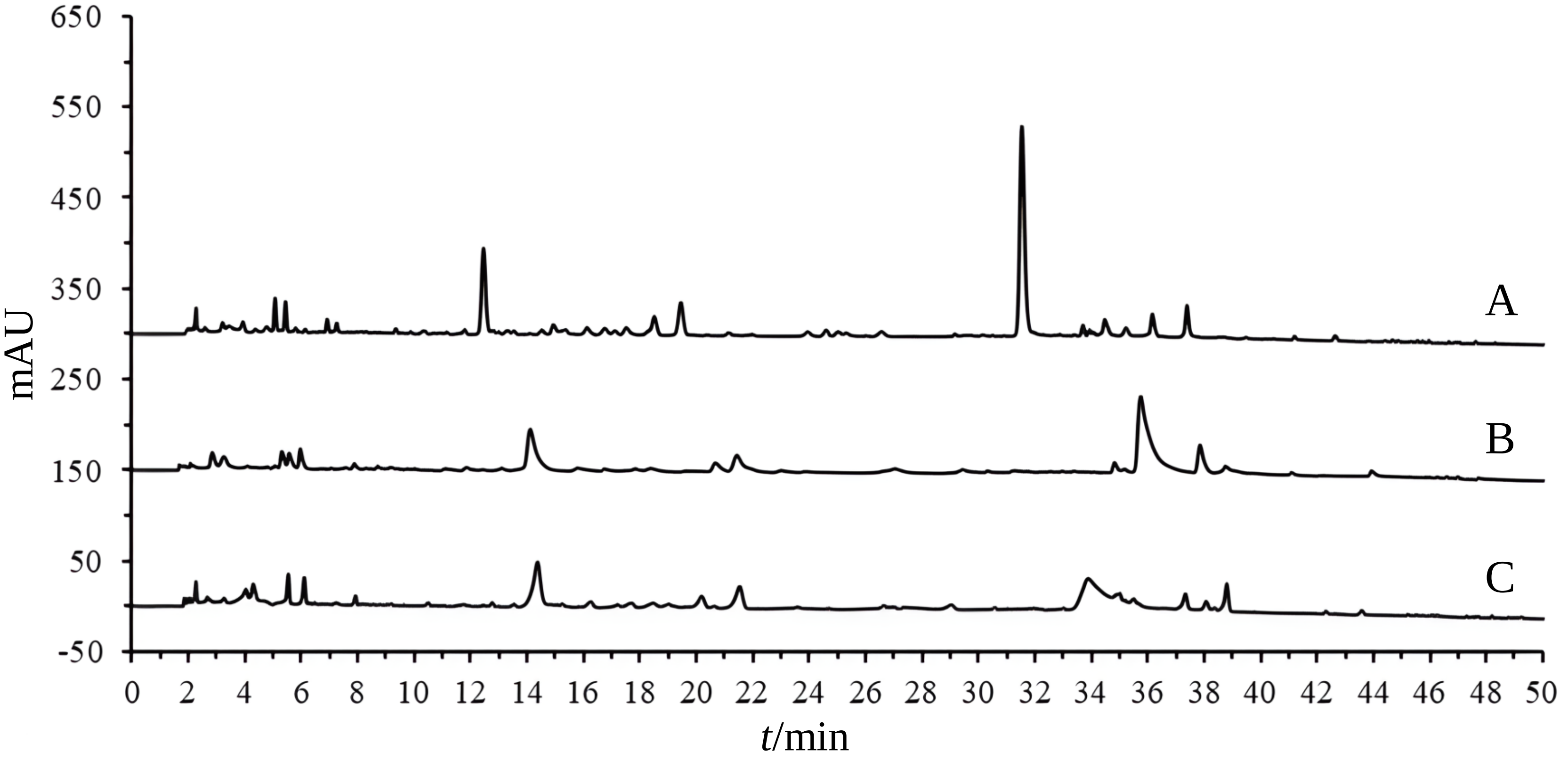

Figure S1. Comparative chromatograms: Thermo Hypersil GOLD aQ (4.6 mm × 150 mm, 5 µm) (A), Agilent ZORBAX SB-AQ (4.6 mm × 150 mm, 5 µm) (B), and Welch Ultimate AQ-C_{18} (4.6 mm × 150 mm, 5 µm) (C).

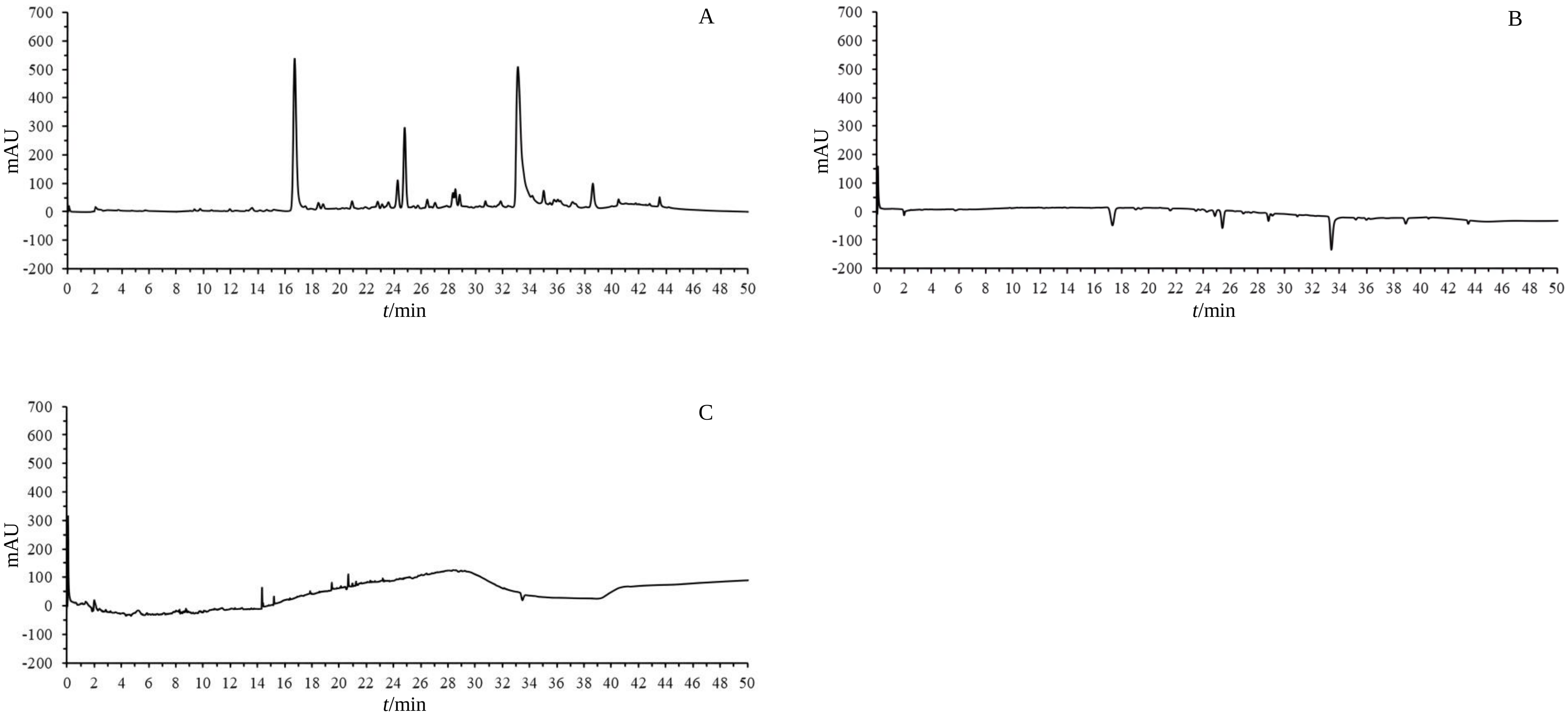

Figure S2. Comparison of chromatograms from online antioxidant systems: Ferric reducing antioxidant power (FRAP) (A), 2,2-azino-bis-3-ethylbenzothiazoline-6-sulphonic acid (ABTS) (B), and 2,2-diphenyl-1-picrylhydrazyl (DPPH) (C).

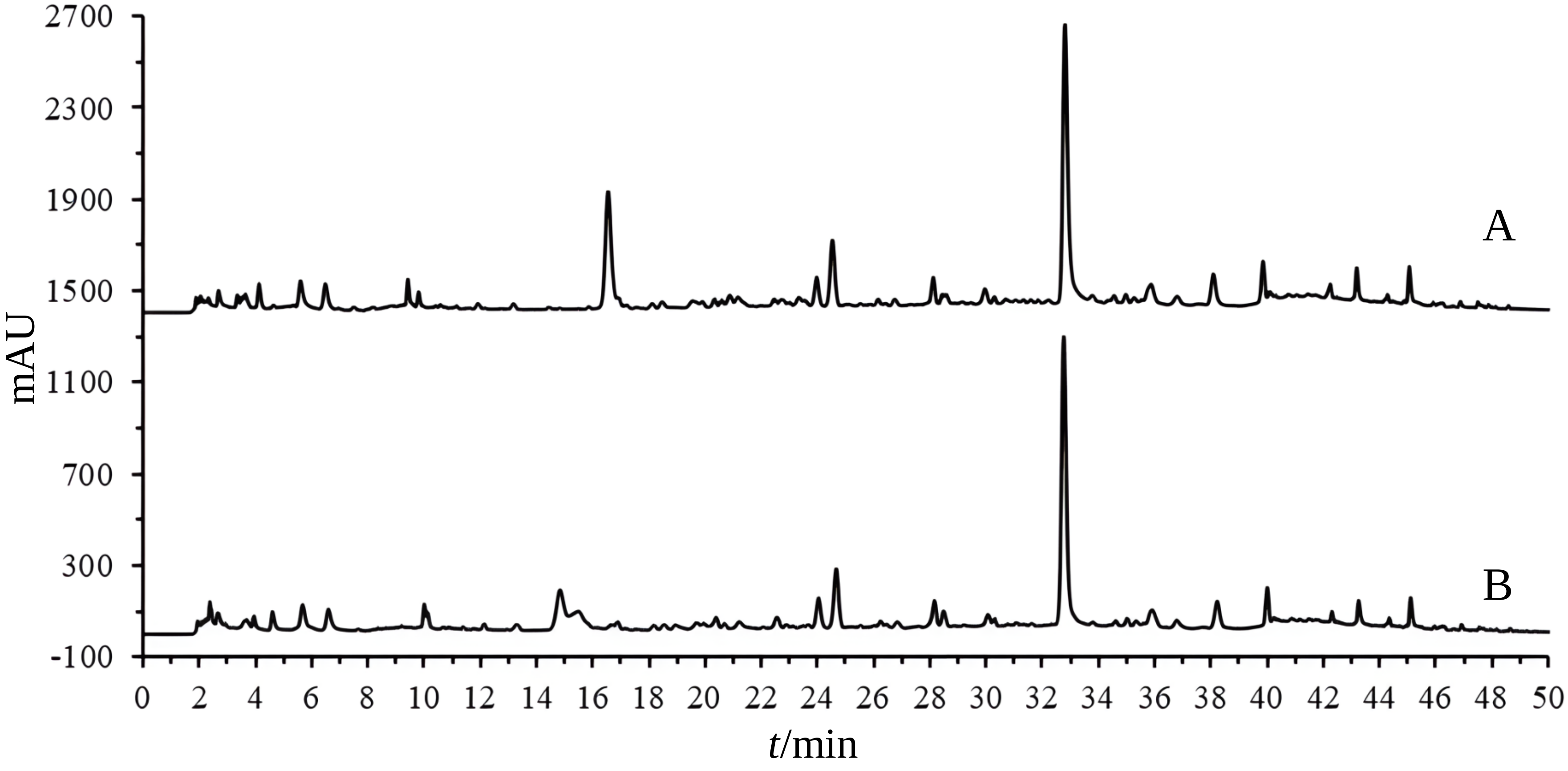

Figure S3. Chromatograms of different dispersants: Acid-washed diatomite (A), and diatomite (B).

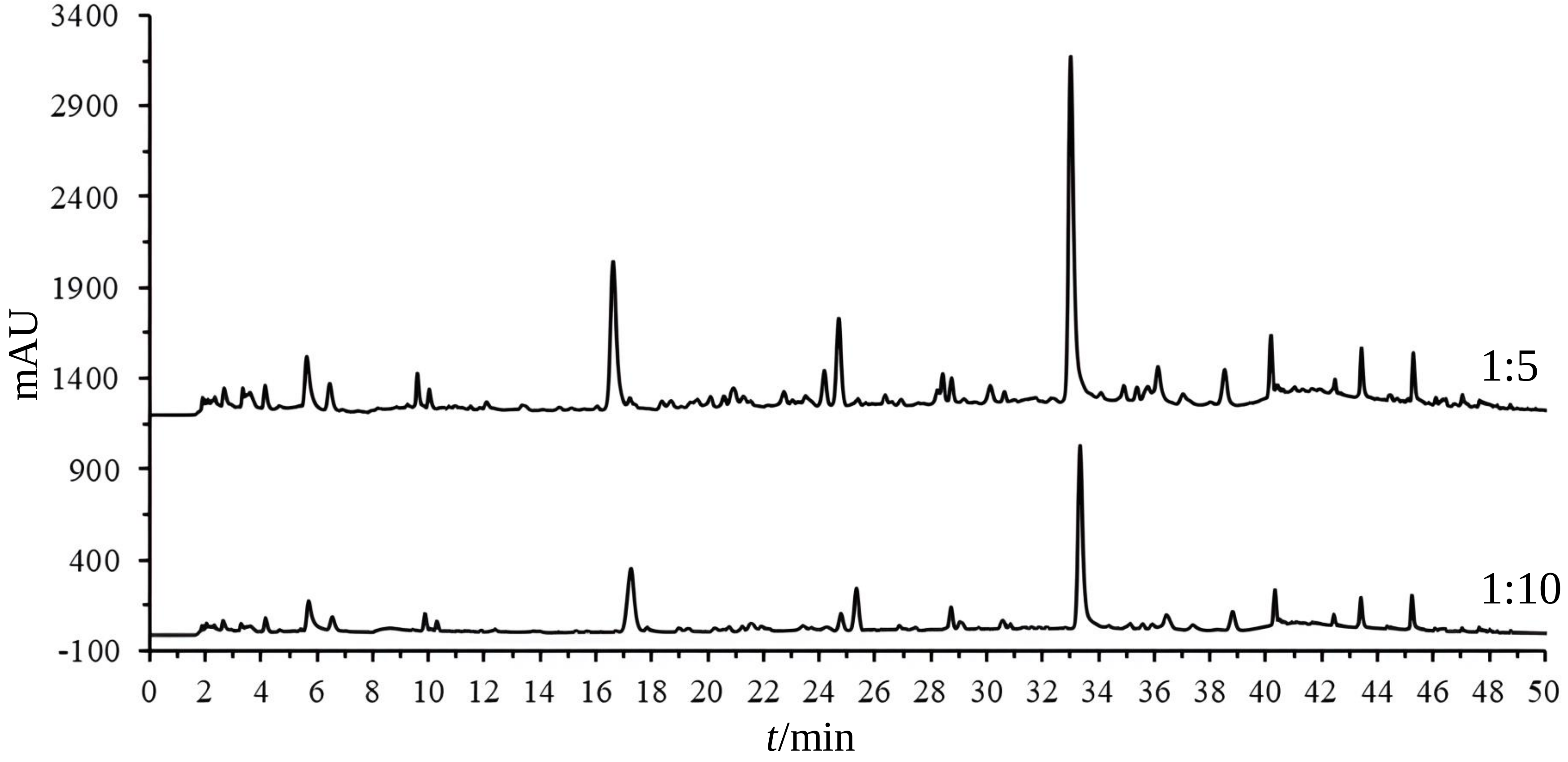

Figure S4. Chromatograms of different ratios (dandelion aerial part to acid-washed diatomite).

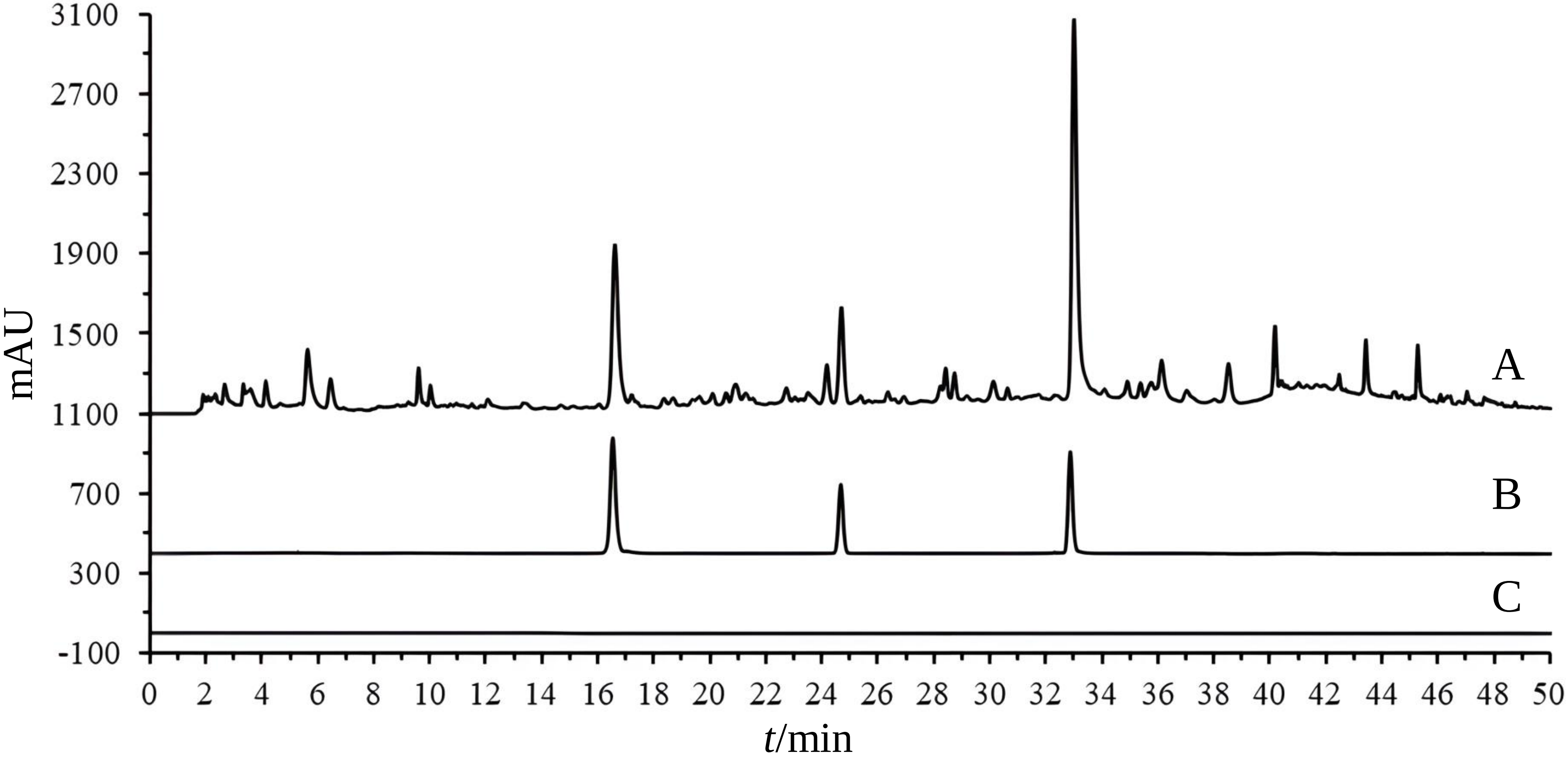

Figure S5. Chromatograms of dandelion aerial part (A), standard solution (B), and blank solution (C).

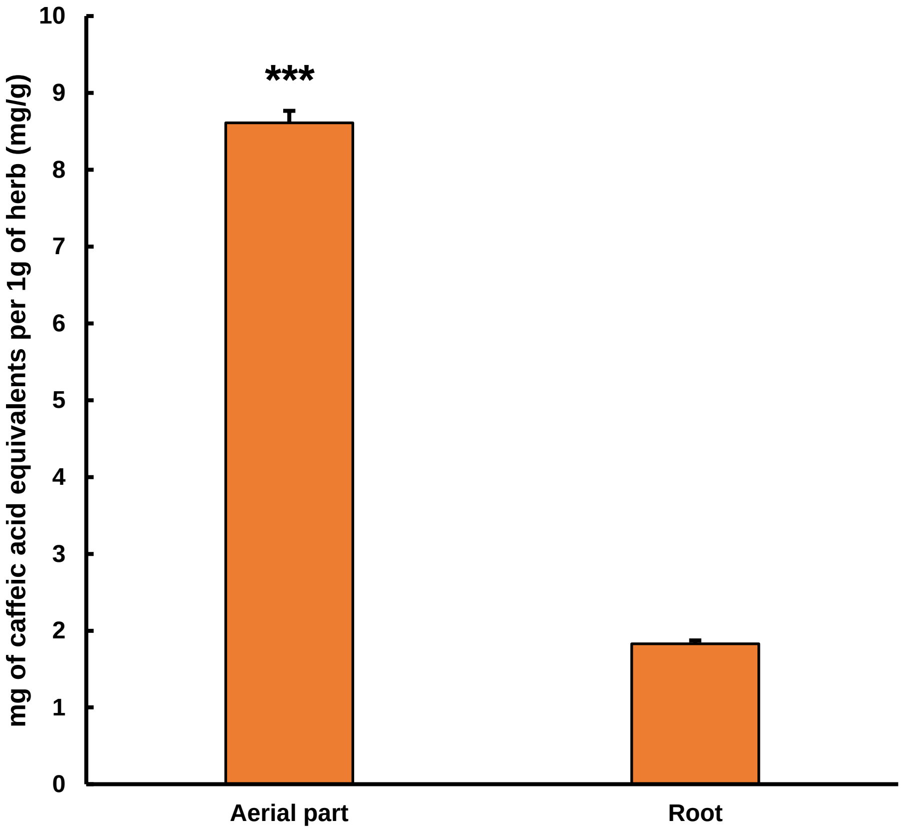

Figure S6. Offline antioxidant analysis of dandelion aerial parts and roots ($n = 3$; mean ± standard deviation). Aerial part: 8.61 mg/g; Root: 1.83 mg/g. *** $p < 0.001$ versus the root, evaluated using Student's t-test.
